# Strategies for Cancer Immunotherapy Using Induced Pluripotency Stem Cells-Based Vaccines

**DOI:** 10.3390/cancers12123581

**Published:** 2020-11-30

**Authors:** Bruno Bernardes de Jesus, Bruno Miguel Neves, Manuela Ferreira, Sandrina Nóbrega-Pereira

**Affiliations:** 1 Department of Medical Sciences and Institute of Biomedicine—iBiMED, University of Aveiro, 3810-193 Aveiro, Portugal; brunob.jesus@ua.pt (B.B.d.J.); bruno.neves@ua.pt (B.M.N.); 2 Center for Neuroscience and Cell Biology (CNC), University of Coimbra, UC Biotech, Biocant Park, 3060-197 Cantanhede, Portugal; 3 Champalimaud Research, Champalimaud Centre for the Unknown, Champalimaud Foundation, 1400-038 Lisboa, Portugal; 4 Instituto de Medicina Molecular João Lobo Antunes, Faculdade de Medicina, Universidade de Lisboa, Av. Professor Egas Moniz, 1649-028 Lisboa, Portugal

**Keywords:** iPSCs, cancer, reprogramming, immunotherapy, neoantigens, vaccines

## Abstract

**Simple Summary:**

Effective cancer immunotherapies, with the objective to boost tumor-specific immune responses, is a game-changer in personalized cancer treatment. In immunotherapy, the immune system is exploited to recognize and destroy cancer cells, and this is only possible if a full recapitulation of tumor specific antigens complexity is achieved. Patient-derived induced pluripotent stem cells (iPSCs) share several characteristics with cancer (stem) cells (CSCs). The exploitation of iPSCs as a source of tumor- and patient-specific antigens guiding the immune system against cancer has been addressed recently in mice. Here, we will debate novel findings on the potential implication of cellular reprogramming and iPSCs plasticity for the design of novel cancer immunotherapeutic strategies.

**Abstract:**

Despite improvements in cancer therapy, metastatic solid tumors remain largely incurable. Immunotherapy has emerged as a pioneering and promising approach for cancer therapy and management, and in particular intended for advanced tumors unresponsive to current therapeutics. In cancer immunotherapy, components of the immune system are exploited to eliminate cancer cells and treat patients. The recent clinical successes of immune checkpoint blockade and chimeric antigen receptor T cell therapies represent a turning point in cancer treatment. Despite their potential success, current approaches depend on efficient tumor antigen presentation which are often inaccessible, and most tumors turn refractory to current immunotherapy. Patient-derived induced pluripotent stem cells (iPSCs) have been shown to share several characteristics with cancer (stem) cells (CSCs), eliciting a specific anti-tumoral response when injected in rodent cancer models. Indeed, artificial cellular reprogramming has been widely compared to the biogenesis of CSCs. Here, we will discuss the state-of-the-art on the potential implication of cellular reprogramming and iPSCs for the design of patient-specific immunotherapeutic strategies, debating the similarities between iPSCs and cancer cells and introducing potential strategies that could enhance the efficiency and therapeutic potential of iPSCs-based cancer vaccines.

## 1. Introduction

Metastatic solid tumors remain an unresolved clinical challenge and one of the leading causes of death. Tumor immunosurveillance is widely recognized as an important mechanism to reduce cancer incidence by the elimination of precancerous lesions. Tumor establishment and progression involve the proliferation of hypo-immunogenic cells that manage to evade the immune system by exploiting immune checkpoints, such as the programed cell death 1 (PD1) PD-ligand 1 (PDL1) axis and cytotoxic T lymphocyte antigen-4 (CTLA-4) [1]. 

The use of the patient’s immune system to eliminate neoplastic cells has long been postulated [2]. However, only the recent increasing understanding of how cancer interacts with the immune system has allowed the proposal of successful cancer immunotherapeutic approaches, creating widespread interest and high expectations in the field [3,4,5]. Immunotherapeutic strategies that successfully train the immune system to efficiently target tumors include (1) unlocking immune checkpoints to achieve tumor recognition and (2) priming the immune system to use a wider repertoire of antigens presented on tumor cells. The latter includes chimeric antigen receptors (CAR) T-cell therapy, where T cells are genetically engineered to produce artificial T-cell receptors, showing impressive clinical outcomes in treating patients with relapsed or refractory B cell malignancies [6,7,8]. Although CAR-T cells have been successfully used even in tumors unresponsive to conventional therapies, many questions limiting its clinical application still need to be addressed. For instance, CAR-T therapies may lead to acute cytokine release syndrome, issues related with long-term safety, acquisition of resistance and lack of clinical efficacy in solid tumors, issues that need to be refined [9,10]. Moreover, several issues persist with the usage of T-cells, including loss of tumor antigen expression, off-target toxicity and the possibility of in vivo transformation, amongst others [8,11,12]. 

Efficient tumor antigen presentation and stimulatory signals by antigen-presenting cells (APCs) is fundamental for a sustained activation of cytotoxic T and B-cells, and crucial for the success of any immunotherapeutic approach [3,13,14,15,16]. Indeed, many tumors present reduced self-antigen presentation due to decreased expression of major histocompatibility complex (MHC)-class 1 molecules, which results from the strong selective pressure imposed on tumor cells in order to evade immunity during early tumorigenesis [17]. Cancer heterogeneity, and the presence of undifferentiated cancer (stem) cells (CSCs), imposes additional barriers with the selective expression of antigens by different cancer cell populations within a tumor [18]. Although clonal neo-antigen burden is associated with improved response to immunotherapy, it is currently challenging to design improved immunotherapeutic approach as tumor samples are often inaccessible [19]. Additionally, novel mutations or clonal selection may lead to the resistance of current immunotherapeutic strategies targeting unique antigens. Thus, a major challenge for cancer immunotherapy is the need to develop more effective strategies since, to date, immunotherapy only works in a subset of cancers and only a fraction of patients with cancer respond to immunotherapy 

Here, we will unveil the potential application of cellular reprogramming and induced pluripotent stem cells (iPSCs)-based cancer vaccines as novel strategies to tackle tumor heterogeneity. Although out of scope of the present review, it is worth to mention that besides direct use of iPSCs as a novel source of cancer antigens, great efforts have been put on the reprogramming of iPSCs into tumor-specific T cell, dendritic cells (DCs), immature natural-killer cells (iNKs,) and gama-delta T cells (γδT) that are then applied in adoptive antitumor immunotherapies [20,21,22,23,24,25,26,27,28]. 

## 2. Cellular Reprogramming Is Prone to Errors 

In 2006, Yamanaka and colleagues challenged the cell differentiation dogma, by demonstrating that a combination of the four transcription factors (TFs) Oct3/4, Sox2, Klf4, and cMyc were enough to reprogram differentiated mouse fetal and adult fibroblasts into iPSCs [29], and one year later, human iPSCs were generated [30,31]. These cells showed characteristics of embryonic stem cells (ESCs) including morphology, ESC-specific gene expression and the ability to form all three embryonic germ layers as depicted by teratoma formation in SCID mice and contribution to chimeric mice [29]. Despite being a major hallmark in cell biology and providing unprecedented opportunity for cell therapy, several challenges still persist that limit their current applications, including their inherent properties of tumorigenicity, immunogenicity, and heterogeneity [32].

iPSCs reprogramming is a very inefficient process leading to a heterogeneous population of cells either failing or reaching only partial reprogramming [33]. Additionally, it is not uncommon that reprogramed iPSCs present mixed response to differentiation stimuli, jeopardizing their potential application [34,35,36]. Both genetic and epigenetic variability may contribute to iPSCs heterogeneity [37], as well as recurrent genetic aberrations [35,38,39,40,41]. Amongst the epigenetic failures is the lack of capacity to fully reset their somatic memory, or to fully establish a new somatic identity [34,36,42,43,44]. Supporting this observation is the common detection of imprinting defects in iPSCs, some potentially halting iPSCs characteristics [41,45,46,47,48,49,50]. Regarding the genetic alterations, iPSCs may reflect the donor genetic background. Indeed, it has been demonstrated that donor genetic material accounted for more functional differences between iPSCs, than the cell type [51]. Indeed, Kilpinen and colleagues [52] described the systematic generation of 711 iPSC clones derived from 301 healthy individuals, outlining that 5–46% of the variation between different iPSCs phenotypes arise from differences between individuals. Although some of the specific traits were associated with pluripotency, others were potentially associated with pathology and tumorigenicity. More recently Salah Mahmoudi and colleagues [53] show that fibroblast from old mice exhibit increased variability during reprogramming. By performing multi-omics profiling of fibroblast cultures from young and old mice, Mahmoudidi revealed the existence of ’activated fibroblasts’ that secrete inflammatory cytokines, more prone to reprogramming. 

Other sources of heterogeneity include the selection of clonal iPSCs carrying specific mutations or the acquisition of mutations during the reprogramming process. iPSCs derivation starts from a few cells and variability was shown to be related with pre-existing variation in the original somatic cells, either contributing to higher or lower reprogramming rates [38,54,55,56,57,58,59]. Several patient-specific somatic mutations impact on iPSCs reprogramming [60], raising important issues when patient-derived iPSCs are required. Those include dysfunction of the mitochondrial respiratory chain [61,62] or chromosomal abnormalities [63,64]. Furthermore, during the in vitro reprogramming process, iPSCs may acquire (additional) somatic mutations which could relate to the reprogramming strategy. iPSCs derivation does not only correlates with pre-existing mutations but could also lead to “de novo” mutations [51,65]; due to the complexity of the reprogramming process is was verified that iPSCs acquire more point mutation than ESCs. One example is the oxidative stress linked mutations acquired during cellular reprogramming [66,67].

As mentioned, the reprogramming process or the subsequent expansion in culture represent additional source of iPSCs heterogenicity. It was previously shown that 17 different human ESCs lines maintained in different laboratories present 843 copy number variations (CNV) [68]. Interestingly, several of the genes detected within CNV sites had altered expression and were functionally linked to cancer. These results support the hypothesis that cellular heterogeneity may lead to the acquisition of cancer like properties. One example linking iPSCs to cancer biogenesis is the abnormal expression of TERT, which activity is mainly restricted to stem cells in the adult tissues [69,70,71]. As iPSCs can be generated from somatic cells, they show great potential for developing patient specific disease modeling, which can be used to study the underlying mechanisms and novel treatments for cancer [72].

Similarly, in vivo reprogramming is also prone to heterogeneity. In vivo iPSCs have an unprecedented capacity to form embryo-like structures, including the three germ layers of the embryo and extraembryonic tissues, still only a small percentage of cells could give rise to such structures [73]. Of noteworthy, and using an assay where GFP-expressing iPSCs and ESCs were microinjected into the morula and the resulting blastocysts were examined, Abad et al observed that iPSCs generated in vivo contributed to the trophectoderm with a remarkable efficiency (56%), which was in contrast to ESCs (0%) [73].

### iPSCs Share Similarities with Cancer Stem Cells

CSCs are subpopulations of cancer cells with stem cell features as self-renewal ability and multi-lineage differentiation that drive tumor growth and heterogeneity. Importantly, CSCs have been invoked as the main drivers of metastatic dissemination, relapse and therapeutic resistance [74,75]. Malignant and metastatic tumors, which evade immune clearance, show a higher incidence of cancer-specific somatic mitochondrial DNA (mtDNA) mutations in protein-coding or RNA-coding regions [76] and expression of stemness genes [75], suggesting that lower immune clearance is associated with increase cancer-specific mutations and expression of stemness markers. Similarly, to ESCs, iPSCs also share a number of characteristics with CSCs including self-renewal and proliferation, expression of stem cell markers and altered metabolism. The similarities between fetal development and cancer has long been recognized, following the discovery of oncofetal proteins (such as α-fetopotein, carcinoembryonic antigen, human chorionic gonadotropic, for a review see [77]), which are tumor-associated antigens (TAAs) expressed during embryonic development and re-acquired in adults during cancer [78]. Human iPSCs share genetic and transcriptomic signatures with cancer tissues, including several cancer-related genes and more than 100 human TAAs and tumor-specific antigens (TSAs), which are protein markers that can be recognized by the immune system [79,80].

In contrast to differentiated cells, cancer cells and ESCs retain the ability to re-enter the cell cycle and proliferate and it does not come to a surprise that common metabolic strategies that fuel anabolic cell growth are adopted. Cancer cells and ESCs present increased glycolytic flux which sustains high rates of cellular proliferation by rapidly generating adenosine triphosphate (ATP), whereas mitochondria is mainly used for anabolic production, a phenomenon known as the Warburg effect [81,82,83]. However, recent evidence suggests that energetics of slow-cycling and therapy-resistant CSCs differ from the bulk proliferative tumor, that largely rely in glycolysis. CSCs present an increased dependency in OXPHOS and mitochondrial respiration and this vulnerability can be exploited for tumor eradication [84]. For ESCs, the main metabolic program adopted and energetic fuel used for ATP production seems to rely on the pluripotency state [81]. When compared to primed, naive ESCs present elevated mitochondrial function, increased OXPHOS, and a lower level of glycolysis whereas primed ESCs present higher mtDNA copy number and more mature mitochondria [81,82].

As for oncogenesis, cellular reprogramming to pluripotency induces a global metabolic remodeling that leads to a progressive transition from somatic oxidative metabolism to glycolysis, with an increase in glycolytic rate and lactate production together with a decrease in cellular respiration and other mitochondrial adaptions [85,86]. Metabolites function goes beyond serving as substrates for energy production and anabolic growth and can determine the balance between CSCs and ESCs self-renewal and differentiation. Many metabolic intermediates (as acetyl-CoA, S-adenosylmethionine, α-ketoglutarate) serve as obligatory substrates or cofactors for chromatin-modifying enzymes, impacting epigenetic regulation and gene expression [82]. For instance, epidermal CSCs avoid epigenetically driven differentiation by shutting down endogenous serine synthesis and becoming dependent on extracellular serine [87]. De novo serine synthesis stimulates α-ketoglutarate-dependent dioxygenases that remove the repressive histone modification H3K27me3, activating differentiation and blocking squamous cell carcinoma growth [87]. Lipid metabolism is also linked to stem cell pluripotency. Fatty acid synthesis is critical for ESCs pluripotency and cellular reprogramming into iPSCs [88]. Likewise, stimulation of de novo lipogenesis by lipid deprivation induces a naive-like state in human PSCs by enhanced histone acetylation and decreased DNA methylation which mimics ERK inhibition [89]. These studies highlight the potential of nutrient manipulation as a strategy to induce metabolic-driven epigenetic and gene expression changes underlying (CSCs and iPSCs) stem cell fate decisions.

For normal or CSCs to maintain their stemness, they must balance both intrinsic and extrinsic signals, maintaining proliferation while inhibiting unwanted differentiation and bypassing immune recognition. Epigenetic regulators are effectors of these inputs with alterations often leading to cell death, or oncogenic transformation and growth [90,91]. Cancer cells are genetically and epigenetically plastic, having the potential to regain benign cell functions via re-expression of lineage-specific genes [90,92]. Recently iPSCs have been proposed as a novel method for studying carcinogenesis through cancer cell reprogramming. A number of human cancer cell lines and primary samples have been used to generate iPSCs, in an attempt to recapitulate the mechanisms underlying cancer development and as a strategy to induce transition from malignancy to benignity [6,90,93]. Since the initial description of reprogramming using the four Yamanaka TFs [29,30], several modifications in the reprogramming protocol aiming at increasing efficiency have been described. Many of those, act commonly in cancer-associated pathways and may contribute to tumor progression, such as down-regulation of the tumor suppressors p53 or p16/nk4a-ARF [94,95]. In line with this, we and others have previously demonstrated that Zeb2, a factor involved in epithelial to mesenchymal transition (EMT) and metastatic potential [96,97,98], is an important barrier limiting the reprogramming of aged fibroblasts [99]. As for CSCs, iPSCs have the potential for infinite proliferation and in certain scenarios, tumorigenic properties have been attributed. Of notice, all of the 4 TFs originally included in the Yamanaka cocktail have proved oncogenic potential [32], especially c-Myc which is one of the most frequently mutated genes in human cancers. Indeed, approximately 20% of the chimeric mice offspring made by iPSCs-derived from 4TFs reprogramming developed tumors attributable to reactivation of the c-Myc transgene [100]. Besides, amplification of genomic and mtDNA mutations during the extensive in vitro iPSCs culturing may represent another source of tumorigenicity and expression of immunogenic proteins [101,102]. Similarly, trophectoderm markers detected in in vivo iPSCs are also linked to the pathobiology of cancer (e.g., Fgfr2) [73]. 

CSCs have the ability for both self-renewal and differentiation and CSCs survive chemotherapy have been shown to be able to re-establish tumors [75,103]. While its origin is not fully known, it has been suggested that CSCs could arise as a result of mutations in stem, transient-amplifying, differentiated or cancer cells leading to acquisition of malignant and/or stem cell properties [92]. Moreover, a number of studies also suggest the existence of plasticity within cancer and stem cell’s compartments [92]. For instance, in breast cancer, normal and CSCs-like cells can arise de novo from more differentiated cell types revealing that mammary stem cells encompass bidirectional interconversions between stem and non-stem compartments [104]. Artificial (or in vitro) cellular reprogramming has also been widely compared to the biogenesis of CSCs [90]. Transitory induction of the 4TFs in mice is able to produce in vivo reprogramming within tissues with formation of teratomas and circulating iPSCs in the blood [73]. Whether reprogramming of somatic differentiated cells into iPSCs is a (naturally) occurring process in vivo and can contribute to cancer and stem cell populations remains to be elucidated.

## 3. iPSCs as Whole-Cell Cancer Vaccines

Our growing knowledge of tumor immunobiology, which is reflected by the constant increase complexity of the immunoediting concept has led, in the last decade, to a paradigm shift in the treatment of numerous cancers [105,106,107]. Blockade of the immune checkpoints represent a remarkable breakthrough in cancer immunotherapy whereas other strategies, such as TAAs/TSAs and DCs vaccines, start to rise as valid contributes to push forward this thrilling field [108]. Here, autologous whole tumor cells vaccines seem to address the tumor heterogeneity caveat. However, this approach requires access to sufficient viable biopsy material and can only be used in a therapeutic setting, without possibility to design prophylactic strategies. 

As previously detailed, cancer transformation is coupled to the appearance of stem-like features, driving the idea that stem/embryonic tissues may harbor the same antigens present in tumors. In this context, ESCs and iPSCs have been extensively tested in anti-tumor immunization protocols [79,109,110]. The seminal experiments of George Schöne demonstrating that immunization with embryonic tissue generates specific antitumor responses date back a century ago [111]. Since then, numerous pre-clinical studies have shown that vaccination with embryonal material prevent not only the growth of transplantable tumors as well as tumorigenesis caused by viral and chemical agents (reviewed in [112]). Nevertheless, ethical constrains associated with the accessibility to ESCs, the reduced repertoire of matching cancer antigens and unwanted immune responses generated by human leukocyte antigen (HLA) incompatibility in allogenic vaccine settings, limited its clinical application [113]. In this regard, autologous iPSCs-based vaccines may address these limitations providing an alternative strategy. The first report of the use of iPSCs as a whole-cell cancer vaccine comes from Li and collaborators, where they compare the efficacy of human iPSCs and ESCs lines as immunizing agents in a transplantable mouse colon carcinoma model [114]. Although vaccination with both human ESCs and iPSC lines induced significant expansion of tumor-specific IFNγ- and IL-4-producing T cells, only human ESC cells inhibited tumor growth. The heterogeneity of oncofetal antigens expression as well as the accumulation of myeloid-derived suppressor cells (MDSCs) in iPSCs-immunized groups were proposed as possible explanations for the distinct anti-tumor protection conferred by iPSCs and ESCs [114] (Table 1). Interestingly, no evidence of autoimmunity was observed, suggesting that the antigens present in ESCs are different from the antigens presented in adult stem cell niches. 

One of the first evidences that autologous iPSCs could elicit an immunogenic response was presented by Zhao et al., where transplantation of immature C57BL/6 (B6)-derived iPSCs (reprogrammed from mouse embryonic fibroblasts) induced T cell infiltration, tissue damage and regression of teratomas in syngeneic recipient mice [115] (Table 1). In this study, T cell infiltration was observed in most teratomas formed by the B6 iPSCs in syngeneic mice, some of which also exhibit tissue necrosis and an apparent regression at 40 days post-implantation [115], supporting that iPSCs are immunogenic in syngeneic recipients.

The concept of iPSCs-based anticancer vaccine was further refined by Kooreman by using irradiated autologous iPSCs in combination with the TLR9 agonist, CpG oligodeoxynucleotide [80] (Table 1). The use of autologous iPSCs allowed to minimize alloimmunity stimulated by MHC mismatches and the addition of the immunostimulatory adjuvant CpG promoted the maturation of APCs such as DCs. In a preventive vaccination setting, injection of irradiated autologous iPSCs plus CpG resulted in the production of tumor-reactive antibodies, expansion of effector/memory helper T cells and mature DCs as well as in a significant decrease in CD4^+^CD25^+^FoxP3^+^ regulatory T cells (T-regs). This favorable CD8^+^ T/T-regs ratio in vaccinated mice resulted in the rejection of transplanted breast cancer, melanoma, and mesothelioma tumor cells [80]. Analysis of early intra-tumor infiltrates revealed increased numbers of vaccine-elicited B and T clones expressing IL-2, IL-4, and IL-5. Moreover, adoptive transfer of T cells from immunized animals to naïve recipients conferred tumor protection, providing clear evidence that the observed effect was being mediated by T cells. When tested in a clinically relevant scenario (i.e., therapeutic administration in animals bearing established melanomas), the iPSCs + CpG vaccine failed to reduce tumor growth. Recently, another study reported that iPSCs therapy was ineffective in reducing melanoma tumor growth probably due to the established immunosuppressive microenvironment [116]. However, as an adjuvant therapy after melanoma resection, iPSCs vaccines reactivated the immune system in eliminating traces of melanoma cells. This rejection was mediated through IL-4 expressing B-cells, TNF-α-expressing CD11b^+^GR1^hi^ myeloid cells, and a reduction of tumor-promoting Th17 cells [80]. This seminal observation created a great enthusiasm given that it showed iPSCs as a TSAs- and TAAs source, readily available at time of tumor resection, allowing to prime and reactivate antitumor immunity and preventing relapses.

Additional studies corroborated the potential of iPSCs-based anticancer vaccines in multiple tumor models. A vaccine composed of Hyper-IL6 (H16) gene-modified melanoma cells admixed with allogenic iPSCs was shown to cause superior extension disease-free survival, and long-term overall survival in melanoma-challenged C57BL/6 mice when compared to modified cells alone or H16 modified cells plus ESCs [117] (Table 1). Mice vaccinated with mixed-iPSCs vaccine shown, in the injection site and spleen, increased numbers of inflammatory monocytes, activated CD4^+^ T cells and memory CD4^+^ T and CD8^+^ T cells, while there was a decrease in the percentage of MDSCs. iPSCs immunization also led to increased percentages of inflammatory monocytes, DCs and NKs in the tumor microenvironment (TME) as well as superior levels of IFN-γ and IL-12p70, causing concomitantly a reduction of infiltrating Tregs [117]. Moreover, Gabka-Buszek and colleagues demonstrated the effectiveness of melanoma vaccines composed of tumor cells mixed with melanoma stem-like cells or iPSCs. In particular, a vaccine containing syngeneic iPSCs and B16F10 cells, significantly inhibited tumor growth and increased mouse survival [117]. 

Additionally, in a humanized-mice model of lung cancer, vaccination with iPSCs + CpG was shown to increase the percentage of splenic APCs and cytotoxic T cells, circulating effector/memory CD4^+^ and CD8^+^ T cells and tumor-infiltrating CD8^+^ T cells, while decreasing Tregs [118] (Table 1). This immune landscape supported an effective suppression of tumor growth, and the central role of tumor antigen-specific T cells was demonstrated by the protection conferred through the adoptive transfer of spleen T cells from the vaccine preimmunized mice to unvaccinated recipients. The protective immunity elicited by iPSCs was proposed to be due to the gene expression pattern shared between iPSCs and lung adenocarcinoma stem cells [118]. 

Although the differentiation of iPSCs is reported to result in loss of immunogenicity [80,120,121], iPSCs-derived pancreatic tumor cells were recently proposed as possible whole cell-vaccines to prevent or delay pancreatic ductal adenocarcinoma (PDAC) in KPC transgenic mice [119] (Table 1). Tumor cells were created by in situ gene editing of healthy fibroblast-derived iPSCs via introduction of *Kras* and *p53* mutations. In contrast to nontransformed iPSCs, the transcriptome of reprogramed cells revealed highly similar gene expression profiles to transgenic mouse-derived tumor cell lines, validating the model as a source of TAAs and TSAs. To increase the immunogenicity, iPSC-derived pancreatic tumor cells were infected with adenovirus or vaccinia virus prior inoculation. The prophylactic vaccination of KPC transgenic mice with adenovirus-infected reprogramed iPSCs followed by boosting with the vaccinia-infected cells was shown to effectively delay PDAC development, significantly prolonging mice survival. Tumor control was associated with increased levels of activated CD8^+^ and CD4^+^ T found in lymph nodes, spleen, and tumor infiltrates. Tumor specific immunity was however lost over time and infiltration of CD8^+^ T cells within tumors was minimal 3 months after immunization [119]. Overall, these studies suggest that autologous iPSCs elicit a specific anti-tumoral response, highlighting the potential use of iPSCs-based vaccines for cancer therapy.

### 3.1. Lessons From ESCs: The Immune Response to Pluripotent Cells 

Pioneer studies using ESCs for cancer vaccination shown evidence of protective effects only when early but not late embryonic tissues were used and this was attributed to the expression of oncofetal antigens [77,112,114]. Although some studies have used ESCs in an allogeneic transfer, due to the limited availability, ESCs cells are more likely to be transferred to an unrelated host which may trigger an alloimmune response. Thus, although indistinguishable from allogeneic responses, an immune response to oncofetal antigens is generated when ESCs are transferred either allogeneically or syngeneically.

The immunogenic response elicited by iPSCs was suggested to contrast with ESCs, where transplantation into syngeneic recipient mice resulted in efficient formation of teratomas without evidence for immune rejection (as indicated by the lack of detectable CD4^+^ T cell infiltration) [115]. ESCs possess immune privileged properties and have the capacity to inhibit immune activation. However, the immunogenicity of ESCs may have been underestimated [122]. Undifferentiated stem cells and derivatives have been shown to induce an immune response in vivo that involves cytotoxic T lymphocytes, helper T cells, and NKs [123,124,125]. Indeed, ESCs express NK cell–activating ligands and are susceptible to NK cell recognition and attack [126,127] although some studies claim that undifferentiated ES cells are resistant to NK cell attack [128,129,130]. 

There are two main classes of polymorphic MHC molecules, human leukocyte antigen (HLA) in humans, expressed by cells that are able to present antigens [131]. While mouse ESCs express mRNA for MHC molecules, but not the corresponding proteins [132,133,134], human ESCs express variable, albeit low levels of HLA class I molecules and almost undetectable levels of HLA class II [125,129,132,133,135,136,137]. As undifferentiated ESCs express low levels of MHC-I and co-stimulatory molecules, transplanted graft-derived antigens may be processed directly by APCs or indirectly through APCs activation of T cells [129,138,139]. In this context, the immune response to transplanted ESCs may involve 3 main developmental stages. The first stage occurs during which recipient MHC class II–restricted CD4^+^ T cells recognize antigens presented by recipient APCs and release proinflammatory cytokines. The second stage occurs when self-restricted CD4^+^ T cells help to generate cytotoxic T lymphocytes that can recognize intact MHC class I molecules. The third stage is antibody response, during which alloantigen-primed CD4^+^ T cells deliver activating signals to B cells.

The low levels of MHC antigens expressed by ESCs are believed to contribute to T cell recognition avoidance even though in vivo ESCs could instead be recognized and susceptible to killing by NKs [126,132,136]. Independently of the immunologic condition of ESCs transfer (syngeneic, allogeneic and xenogeneic), the innate immune response involving NKs plays a critical role against transplanted ESCs. Moreover, MHC expression can be increased during ESCs differentiation and in the presence of cytokines and while this may protect from NKs lysis, it will make cells visible to cytotoxic T cells [50,125,140], suggesting that modulation of MHC expression in PSCs (e.g., inflammatory environment) is a key factor for immunogenicity. 

Heat shock proteins (HSPs) mediate cross-priming via both chaperoning antigenic peptides for cross-presentation to MHC and activating DCs [141]. Undifferentiated human ESCs are enriched with multiple HSPs (e.g., gp96, HSP86, HSP84, GRP78, HSC70) and calreticulin [114], which could promote cross-presentation of antigens to host DCs. While the use of B-cell–deficient mouse models revealed that B cells and antibodies are not critical for the primary immune response to ESCs [142], a strong cell- and humoral-based immune responses was generated when ESCs were used as cancer vaccines. Several studies have shown a broad spectrum of tumor-specific immune response upon ESCs immunization with identification of specific cross-reactive immune response between tumors and ESCs [114,143,144,145]. Whether this reactivity is due to allogeneic responses that indirectly prime T cells to cross-react with multiple oncofetal-antigens or, on the other side, ESCs-expressed oncofetal antigens that trigger a direct and specific immune response remains unclear.

### 3.2. The iPSC-Elicited Immunogenic Response

The mechanism and players involved in the host immunogenic response to transplanted iPSCs remains scarce. The immune rejection response to iPSCs is T cell dependent, supported by the fact that this response was absent in recombination activating gene (RAG) knock-out recipient mice which lack both CD4^+^ helper and CD8^+^ cytotoxic T cells [115]. Moreover, teratomas formed by human iPSCs in humanized-mice reconstituted with autologous immune system indicated that the majority of teratomas contained regions that were infiltrated with human CD4^+^ and CD8^+^ T cells [146]. In contrast to differentiated iPSCs-derivatives, which developed a tolerogenic immune network, undifferentiated iPSCs elicit an immune response associated with high intragraft lymphocytic infiltration and elevated IFN-γ, granzyme-B and perforin cytotoxic effector molecules [120]. 

Apart from T cells, it is likely that iPSCs derived grafts are infiltrated by additional immune cells that might contribute to iPSCs rejection. For instance, iPSCs express NKG2D ligands at the cell surface and are highly susceptible to natural cytotoxic cells [121]. Tumor infiltrating innate lymphoid cells (ILCs) can show an activated phenotype (expression of CD69, CD44, MHCII, and KLRG1) and provide tumor immunosurveillance [147,148]. Evidence suggest that cell transformation expands tissue-resident type-1-like innate lymphoid cells (ILC1ls) and type 1 innate-like T cells (ILTC1s) [149]. This is relevant because ILC1s exhibit NKs-like characteristics and, in the presence of IL15, can express IFN-γ and eliminate malignant cells exerting antitumor functions [150,151]. Also, ILC3s recognize tumor cells and facilitate leukocyte tumor entry, increasing anti-tumor immunity [152]. Given the involvement of ILCs in the TME and the fact that iPSCs express neo/oncoantigens, it is possible that ILCs may potentially invade implanted iPSC grafts as well as being involved in the iPSCs-elicited immunogenic response.

**Figure 1:**
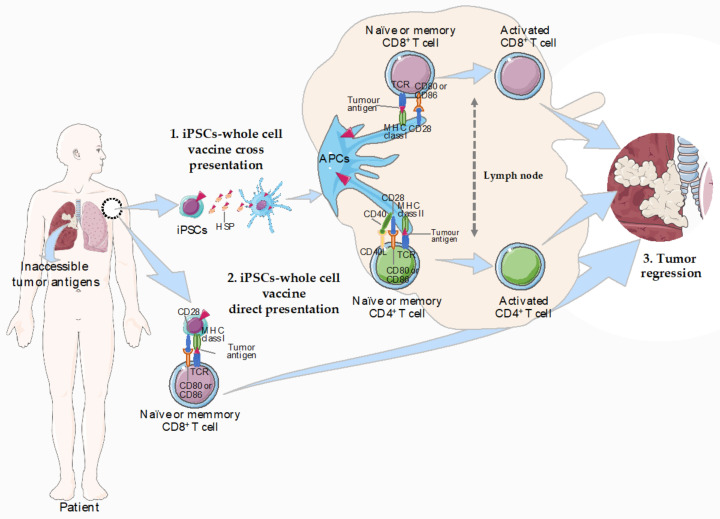
Possible pathways for immunogenic activation in response to autologous iPSCs-whole cell vaccines. In the indirect pathway (1), endogenous APCs uptake antigens in the patient’s body. The expression of HSPs in iPSCs is likely to promote cross-presentation by APCs (e.g., DCs) which are able to capture and process antigens, converting proteins to peptides that are presented on MHC molecules. This is followed by specific binding of a TCR on T cells in draining lymph nodes to its cognate peptide-MHC complex displayed on an APC-like, macrophages or DCs. Two signals are required for T-cell activation. Binding between the MHC/peptide complex on the APC and the TCR for antigen on the T cell is the first signal. The second signal is provided when co-stimulatory molecules (e.g., CD40 or CD80/86 on the APCs and CD40L or CD28 on T cells, respectively) are ligated. In the direct pathway (2), iPSCs express low levels of MHC class I and can directly present peptides to CD8^+^ T cells without the need of APCs. Still, cytokines secreted by activated T cells are necessary as recognition of antigen alone is insufficient to induce full activation of naïve CD8^+^ T cells. As a result of T-cell activation, the APCs or T cells secrete cytokines that drive lineage differentiation into effector killing cytotoxic T cells that can lead to tumor regression (3). Abbreviations: APCs, antigen-presenting cells; DCs, dendritic cells; iPSC, induced pluripotent stem cell; MHC, major histocompatibility complex; TCR, T-cell receptor; HSPs, heat shock proteins. This figure was created using Servier Medical Art templates, which are licensed under a Creative Commons Attribution 3.0 Unported License; https://smart.servier.com.

Donor-derived MHC antigens expressed by transplanted cells normally trigger a T-cell dependent response, which can occur by 2 distinct pathways: (1) T cells interact with processed donor-derived peptides bound to MHC molecules on host-APCs (indirect pathway) [153] or (2) T cells recognize donor peptides displayed by MHC molecules on the surface of the transplanted cells (direct pathway) (Figure 1). Once expressing MHC molecules, transplanted iPSCs can present antigens in a MHC-restricted fashion, either via MHC-I or acting as nonprofessional APCs via MHC class II molecules [154]. Similarly to ESCs, human iPSCs were shown to lack MHC-II while expressing higher levels of MHC-I and b2-microglobulin [135]. Moreover, Suárez-Alvarez et al., provided evidence that transporter associated with antigen processing 1 (TAP-1) and tapasin (TPN), both involved in the transport and load of peptides, are expressed at low levels by iPSCs [155]. The iPSCs-elicited immunogenic response may involve the uptake of dying cells and presentation of iPSCs-derived antigens by specific APCs, as macrophages or DCs (Figure 1). Indeed, increasing evidence supports that iPSCs transplanted cell-specific antigens are processed by the APCs to T cells, indicating an indirect recognition by T cells [156,157,158,159]. For instance, the undetectable immune response to iPSC-derived grafts transplanted under the kidney capsule in mouse was attributed to the lack of functional DCs [160]. On the other side, the expression of MHC-I suggests that iPSCs are able to directly present specific antigens to T cells (Figure 1). Further studies are warranted to clarify the mechanism by which iPSCs-derived cancer vaccines trigger an immunogenic response in the host.

### 3.3. What’s Behind the iPSCs-Elicited Immunogenic Response?

In mice, the immunogenicity of iPSCs was found to be negligible upon differentiation into several cell types and tissues, including skin, bone marrow, hepatocytes, or neurons [161,162]. Indeed, the immunogenicity of iPSCs has been reported to decrease the closer the surface antigen expression profile becomes to that of the parent somatic cells [120]. These observation supports the idea that while terminal differentiation of iPSCs results in loss of immunogenicity and leads to the induction of tolerance [120,121,161,162], the autologous transplantation of undifferentiated iPSC states is highly immunogenic [80,115]. Immunity to stemness genes may be critical for harnessing the immune system against cancer [122,163] and immunogenicity to the TFs used for cellular reprogramming has been reported. The reactivation of Oct4 and Sox2 expression could be at the basis of iPSCs immunogenicity, since both ESCs antigens have been shown to promote T-cell dependent immunogenic responses in healthy and neoplastic conditions [138,164]. Indeed, healthy humans harbor Oct4-specific memory T cells in peripheral blood and the reactivity to Oct4-derived peptides resides primarily in the CD45RO^+^ memory T-cell compartment and peptide-specific IFNγ-secreting CD4^+^ T cells [138]. Oct4 has also been implicated as an oncogene in the pathogenesis of germ-cell tumors (GCTs), a class of tumors that lack MHC II expression [165,166]. In contrast to healthy donors, immunity to Oct4 was detected in only 35% of patients with newly diagnosed GCTs. However, chemotherapy treatment leads to the induction of Oct4-induced immunity in vivo presumably by DCs presentation of Oct4 from dying GCT cells [138]. Whether endogenous Oct4-expressing stem cells pools are actively recognized by T cells and its implication in homeostasis and pathology (cancer, autoimmune diseases) remains to be clarified. Nevertheless, these studies demonstrate the lack of immune tolerance to critical pluripotency antigens in humans and could provide a mechanism for constant immune surveillance against unrestrained growth of pluripotent (normal or cancer) stem cells.

The use of ESCs for anti-cancer immunization raises several concerns for clinical application, including ethical and alloimmunity safety issues. Undifferentiated autologous iPSCs may provide a more accurate and representative panel of patient’s tumor immunogens that non-autologously derived ESCs [80]. This raises the question of whether (viral or non-viral based) reprogramming approaches trigger an abnormal expression of antigens, unexpressed during normal development or differentiation of ESCs, breaking the peripheral tolerance and promoting a T-cell dependent immune response, a scenario resembling the response elicited by TSAs. In the pioneer studies by Zhao et al., several genes abnormally expressed in iPSCs-derived regressing teratomas, including the zymogen granule protein 16 (*Zg16*) and the tumor antigen HORMA domain-containing protein 1 (*Hormad1*), were suggested to contribute directly to the T-cell dependent immune response to B6 iPSCs [115]. The specific expression of these antigens could be attributed to epigenetic modifications [42] or mutations in the coding sequence [38] acquired during the reprogramming process or in vitro culturing, which are absent from ESCs and promote unique immunogenicity of iPSC derivatives. Recently, de novo mtDNA mutations were proposed as a potential source of immunogenic neoepitopes in mouse fibroblasts-derived autologous iPSCs [102]. These mtDNA mutations encode neoantigens that provoke an immune response highly specific and dependent on the host MHC genotype. For instance, mutant *mt-Co1* generates novel cytochrome C oxidase I (Co1) peptides with MHC binding affinity which could elicited IFN-γ and IL-4 responses in mice and this acquired antigenicity persists even after differentiation, suggesting that the response is independent from the iPSCs pluripotency state [102]. Overall, these studies suggest that unique gene expression in undifferentiated iPSCs can induce T-cell dependent immune responses. Understanding the key molecular players and neoantigens that elicit the iPSCs-immunogenic response may allow immune-based prevention and stronger immunogenicity in cancer therapy.

## 4. Potential Strategies to Refine iPSCs-Based Vaccines and Current Limitations

**Figure 2:**
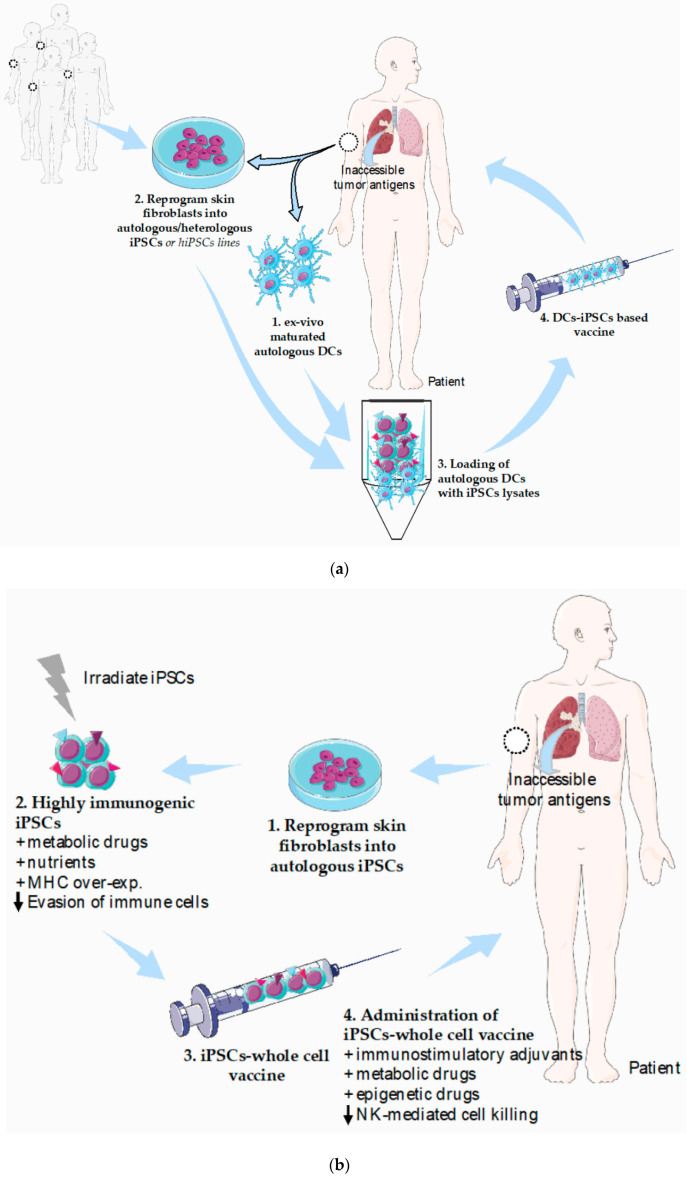
Potential strategies to refine iPSCs-based cancer vaccines. (**a**) Schematic illustration of the combination of iPSCs and DCs-based vaccines and application in cancer patients. Autologous DCs are obtained from peripheral blood (1) followed by adequate ex vivo DC maturation. A sample of skin (2) is used to obtain adult fibroblasts that will be reprogrammed into autologous iPSCs by the introduction of the four Yamanaka transcription factors or alternatively, established syngeneic human iPSCs lines could be used. (3) iPSCs are used to exogenously load autologous DCs ex vivo and the DCs-iPSCs based vaccines will be is administered in the cancer patient (4). (**b**) Schematic illustration of same strategies that could improve the efficacy of autologous iPSCs-whole cell vaccines. Autologous skin-derived fibroblasts are reprogrammed into iPSCs (1) followed by ex-vivo treatments to enhance iPSCs immunogenicity and irradiation for safety (2). iPSCs-whole cell vaccines will be administered into cancer patients (3) in combination with cytotoxic T and APCs immunostimulatory and/or NKs-mediated cell killing inhibitory strategies (4). Abbreviations: APCs, antigen-presenting cells; DCs, dendritic cells; iPSCs, induced pluripotent stem cell; NKs, natural-killer cells. This figure was created using Servier Medical Art templates, which are licensed under a Creative Commons Attribution 3.0 Unported License; https://smart.servier.com.

It has become widely accepted that simultaneous tackling of several cancer hallmarks is key to achieve more effective therapies. In agreement, combining iPSCs-based cancer vaccines with immune checkpoint inhibitors or DC-based immunotherapies represent attractive approaches. Specifically, the combination of iPSCs and DC-based vaccines is of major interest since it solves two major limitations of each individual approach (Figure 2a). In one hand, iPSCs are an easily accessible tumor antigen source that can be used to load ex vivo differentiated DCs, circumventing the need of frequently inaccessible or insufficient tumor biopsy material. On the other hand, administration of autologous DCs exogenously loaded with iPSCs lysates instead of iPSCs whole cells vaccine will minimize concerns related to teratoma formation. Finally, ex-vivo manipulation ensures adequate DC maturation status prior to vaccination, rendering the approach less dependent of the action of immunosuppressed endogenous DCs frequently observed in cancer patients.

Strategies that could target multiple TSAs at once induce a broader spectrum of antitumor immunity and possibly provide more effective and durable protection against cancer [167]. In this regard, autologous iPSCs-based whole cell cancer vaccines circumvents the ethical and safety issues raised by the use of ECSs. Several clinical trials that use human iPSCs for Cancer Immunotherapy are currently ongoing (UMIN Clinical Trials Registry (https://www.umin.ac.jp/ctr/index.htm). Still, from the immune point of view, additional strategies for improving the host immunogenicity to autologous iPSCs-cancer vaccines could be adopted (Figure 2b). Increasing the knowledge on the iPSCs-driven immunogenic response (e.g., tumor antigens, MHC molecules, NK activating molecules) is imperative. Another key issue is to minimize immune evasion of iPSCs-based cancer vaccines. Guarantee should be taken that mechanism of immune evasion used by cancer cells (e.g., CTL-4; PD-1L) are not acquired by iPSCs. There are several strategies that tumor cells employ during immune evasion, some of which include MHC class I structural alterations/downregulation, mutations of Fas or TRAIL and inhibition of T cell receptors and/or development of Treg [168,169,170]. In the context of direct antigen presentation to T cells, modulating MHC expression in iPSCs could be a strategy, as MHC expression can be increased during differentiation of ESCs and in the presence of cytokines, such as IFN-γ [125,140]. Manipulation of HLA expression in stem cells has recently been shown as a promise strategy for the generation of hypoimmunogenic grafts. Thus, in the same way as MHC knockdown in ESCs [171], it might be possible to use MHC-overexpressing iPSCs to potentiate their ability to present neo-antigens (Figure 2b). On the other hand, up-regulation of MHC might decrease the susceptibility of cells to NK cell–mediated killing [121,126]. 

The establishment of an immunosuppressive TME has been suggested to decrease the anti-cancer efficacy of iPSCs-based vaccines [116]. Metabolic adaptations of stromal and immune cells in the TME influence cancer progression [172] and modulation of metabolism can enhance cancer immunotherapy [173]. For instance, combining glycolysis inhibition with adoptive T cell therapy (ACT) is a potential strategy to increase the response of ACT-refractory melanoma patients [174]. During reprogramming of somatic cells into iPSCs, cells undergo a metabolic remodeling that accompanies the remarkable transcriptional and epigenetic changes. Conversely, several metabolic adaptations (e.g., increase in fatty acid synthesis) potentiate the efficiency of cellular reprogramming and promotes a naive-like state in human PSCs [89,175]. Recently, de novo mutations in the mtDNA of iPSCs have been shown to produce immunogenic neoepitopes in mice and humans [102]. This raises several questions and opportunities. Can we modulate nutritional cues and metabolic pathways, including mitochondrial activity, to produce more immunogenic iPSCs for cancer vaccines? The combination of iPSCs with epigenetic drugs (histone deacetylase inhibitor) has been shown to block metastasis in breast cancer [176]. Is metabolic-driven epigenetic modulation able to enhance the acquisition of new neoantigens that elicit a superior iPSCs immunogenic response? Whether selective metabolic adaptations can induce the expression of neoantigens that lead to more immunogenic autologous iPSCs-derived cancer vaccines remains to be explored (Figure 2b).

Ideally, iPSCs would mimic tumor cells in its plenitude. To avoid iPSCs becoming tumorigenic, cells are reprogrammed and grown in vitro and subsequently irradiated previous to implantation in the patient. However, care must be taken regarding teratoma formation and auto-immunity before moving this treatment into the clinical setting. The complexity of this process raises additional limitations and concerns regarding the successful implementation and safety of iPSCs-based cancer vaccines. For a cancer vaccine to be effective, it must be processed by APCs and transported to draining lymph nodes (dLN), where the presented TAAs activate tumor-specific cytotoxic CD8^+^ T cells (Figure 1). The route of iPSC vaccine administration should carefully be evaluated in order to achieve enhanced immunogenic anti-tumor protection as the TME has been shown to determine the immunogenicity of iPSCs [123,160,177,178]. A careful study that correlates the route of iPSCs implantation in the host (intramuscular, subcutaneous, intravenous) with the efficacy of the immune response is warranted in order to achieve major anti-tumor protection. One of the major limitations of cancer immunotherapy addressed through the use of iPSCs is the potential access to TAAs/TSAs, including patient specific and, probably, neo-antigens. Still, iPSCs-based cancer vaccines may share several limitations with the current immunotherapeutic strategies. This may include hypo-responsiveness or hyper-responsiveness scenarios, and limitations in iPSCs generation that hamper vaccine production. Hypo-responsiveness may relate to the intrinsic properties of cancer development to persist and survive. We cannot rule out the long-term inefficacy of iPSC-based vaccines, or a non-iPSCs related efficacy. Indeed, Kooreman and colleagues observed that under defined settings, unspecific activation of the immune system was enough to decrease tumor size, in the absence of a targeted therapy [80]. Regarding hyper-responsiveness it is important to understand which antigens may be mediating iPSCs immunogenicity. Multiple presentation of common and unique antigens may lead to confound responses, including initially tumor-specific targeting, which could eventually evolve to unspecific immune reactions. Indeed, immune-related toxicity may appear as an outcome of PD-1/PD-L1 or CTLA-4 blockage [179,180], which directly halts the immune recognition, or secondarily promotes the acquisition of autoimmunity. The bibliography is limited regarding the capacity of iPSCs or iPSCs derivatives to build long-term responses again own stem cells, amplifying progenitors or tissues. Lastly, despite the great advances, there are still limitations in iPSCs reprogramming and maintenance, particularly for the human naïve state. In summary, and although the current benefits seem to outweigh the risks, we are still far from understanding the long-term impact of moving iPSCs-based cancer vaccines into the clinical setting.

## 5. Conclusions

The development of novel cancer therapies that address unmet medical needs is warranted. A growing body of interest in the impact of cancer immunotherapy is emerging and it may be considered the future of personalized medicine in cancer therapy. Still, the correct antigen loading and tumor targeting is limiting the widespread application and efficacy of current immunotherapeutic strategies. iPSCs share known and unknown TAAs/TSAs, and therefore can potentially prime the immune system against cancer, bypassing the need of tumor collection. We are in the first steps of iPSC-based cancer therapeutic strategies, still it will be interesting to unveil whether patient-specific iPSCs are bona-fide carriers of TAAs/TSAs and the potential use of iPSCs-based cancer vaccines in a prophylactic setting before the appearance of oncogenic transformation. In the light of recent advances on the potential use of iPSCs-based therapies discussed here, we believe that combinatorial interventions (e.g., iPSCs plus checkpoint blockade or DCs-based immunotherapies) may hold great promise to thrive the development of personalized cancer therapies. 

## Figures and Tables

**Table 1:** Immunogenicity responses elicited by induced pluripotent stem cells (iPSCs) whole-cell cancer vaccines in mouse models.

Species	Cell of Origin	Reprogramming Conditions	iPSCs State	Local of Engraftment	Immunogenicity Test	Immune Response	Immunogenic Proteins	Tumor Targeted	Study
*Homo sapiens*	Fetal lung fibroblast line IMR90	Lentivirus expressing 6F ^1^	TTZ1 cell line	sc ^2^	Teratomas	Tumor-specific IFN-γ-and IL-4-producing T cells	Oncofetal antigens	Colon carcinoma	[114]
*Mus Musculus*	MEFs ^3^	Retrovirus expressing 3F ^4^ and 4F ^5^, 4F non-integrative episomal vector	SSEA-1	sc (hind leg)	Teratomas IFN-γ releasing assay in vitro	CD4^+^ helper T CD8^+^ cytotoxic T	Hormad1 Zg16	na ^6^	[115]
*Mus Musculus*	Fibroblasts from FVB, C57BL/6J, and CBA/J mice	Codon-optimized mini-intronic plasmid containing 4F	SSEA-1	sc (flank)	Teratomas	Increase in effector/memory helper T cells, mature DCs, IL-4-expressing B cells, TNF-α expressing myeloid cells; decrease in Tregs and Th17 cells	Oncofetal antigens	Breast cancer, melanoma, and mesothelioma tumor cells	[80]
*Mus Musculus*	MEFs	Nucleofection with plasmid coding 4F	SSEA-1; Epcam, E-cadherin, NANOG, alkaline phosphatase	sc	na	Inflammatory monocytes, activated CD4^+^ T cells, memory CD4^+^ T and CD8^+^ T; tumor infiltrating DCs, NKs, inflammatory monocytes; decrease in Tregs and MDSCs	TAAs ^7^/TSAs ^8^ shared with CSCs ^9^	Melanoma	[117]
*Homo sapiens*	Fibroblasts	na	na	sc	na	Splenic APCs and cytotoxic T cells; circulating effector/memory CD4^+^ and CD8^+^ T cells; tumor infiltrating CD8^+^ T cells; decrease in Tregs	TAAs/TSAs shared with CSCs	Lung cancer	[118]
*Mus Musculus*	Tail-tip fibroblasts	Retrovirus expressing 3F	Endo factors; colony formation assays	sc	IFN-γ releasing assay in vitro	Increase in activated CD8^+^ and CD4^+^ T cells	TAAs/TSAs	Pancreatic ductal adenocarcinoma	[119]

^1^ 6F (Oct3/4/Sox2/Lin28/Klf4/Nanog/c-Myc); ^2^ subcutaneous; ^3^ mouse embryonic fibroblasts; ^4^ 3F (Oct4/Sox2/Klf4); ^5^ 4F (Oct4/Sox2/Klf4/cMyc); ^6^ non-addressed; ^7^ tumor associated antigens; ^8^ tumor specific antigens; ^9^ cancer stem cells.
